# Impact of Epigenetics, Diet, and Nutrition-Related Pathologies on Wound Healing

**DOI:** 10.3390/ijms251910474

**Published:** 2024-09-28

**Authors:** John Hajj, Brandon Sizemore, Kanhaiya Singh

**Affiliations:** 1 Indiana Center for Regenerative Medicine and Engineering, Department of Surgery, Indiana University School of Medicine, Indianapolis, IN 46202, USA; johnhajj@iu.edu (J.H.); brandon.sizemore@cshs.org (B.S.); 2 McGowan Institute for Regenerative Medicine, Department of Surgery, University of Pittsburgh, Pittsburgh, PA 15219, USA

**Keywords:** chronic wounds, wound healing, diet, diabetes, obesity, nutrition, genomics, epigenetics, socioeconomics

## Abstract

Chronic wounds pose a significant challenge to healthcare. Stemming from impaired wound healing, the consequences can be severe, ranging from amputation to mortality. This comprehensive review explores the multifaceted impact of chronic wounds in medicine and the roles that diet and nutritional pathologies play in the wound-healing process. It has been well established that an adequate diet is crucial to proper wound healing. Nutrients such as vitamin D, zinc, and amino acids play significant roles in cellular regeneration, immune functioning, and collagen synthesis and processing. Additionally, this review discusses how patients with chronic conditions like diabetes, obesity, and nutritional deficiencies result in the formation of chronic wounds. By integrating current research findings, this review highlights the significant impact of the genetic make-up of an individual on the risk of developing chronic wounds and the necessity for adequate personalized dietary interventions. Addressing the nutritional needs of individuals, especially those with chronic conditions, is essential for improving wound outcomes and overall patient care. With new developments in the field of genomics, there are unprecedented opportunities to develop targeted interventions that can precisely address the unique metabolic needs of individuals suffering from chronic wounds, thereby enhancing treatment effectiveness and patient outcomes.

## 1. Introduction

The influence of chronic wounds is vast, impacting many aspects of the medical field. Based on 2019 data, 10.5 million United States patients had chronic nonhealing wounds [1]. Medicare cost estimates for acute and chronic wounds range from $28.1 to $96.8 billion, with a variety of pathophysiologies influencing the development of such wounds [2]. Healthcare costs in the US have continued to rise, and in addition to an aging population and increasing incidences of chronic conditions like obesity [3] and diabetes [1], the burden of chronic wounds continues to pose an immense threat to patients and the healthcare system. The situation calls for increased literature on chronic wound formation mechanisms and their prevention [4]. Etiologies underlying the formation of chronic wounds vary. Vascular disease, pressure, diabetes, obesity, ischemia, hypertension, and malnutrition are some of the leading causes of delayed and improper wound healing [5].

Once a chronic wound has formed, the risk of infection increases, further complicating the prognosis and increasing the financial burden and risk of morbidity for the patient [6]. In many cases, an untreated chronic wound results in amputation. Along with infection, diabetes is the most common risk factor for amputation. Between 2010 and 2019 in the US, it was found that approximately 68.6% of all amputations were associated with diabetic wounds [7]. While highly manageable, the burden of amputations is severe, posing significant socioeconomic challenges to the individual and the healthcare system overall. Regarding healthcare costs alone, diabetic-related amputations in the US in 2001 accounted for almost $11 billion, with the number steadily increasing since [8]. For the individual, the challenges of amputation are widespread and long-lasting, ranging from financial instability to mental illness. Girijala et al. found that amputees reported a significantly lower quality of life due to loss of income and repeated episodes of depression and anxiety [9]. Increased mortality rates post-amputation have also been documented. Studies conducted prior to 2010 indicated that patients suffering from diabetes-related amputations had a 5-year survival rate of 40–48% [10]. These reports are clearly a cause of concern for the health and socioeconomic challenges that amputees face daily and further warrant the study of chronic wound prevention and management. The rising prevalence of obesity, diabetes, and other nutrition-related processes in the US prompts the need for further research into how malnutrition, lifestyle, and other socioeconomic factors affect the patient at the molecular and cellular level and how they influence the development and healing of chronic wounds. Through analysis of the current literature, this review seeks to contribute to a deeper understanding of the roles of macronutrients and micronutrients in the stages of wound healing. By investigating the consequences of nutritional deficiency as well as the benefits of supplementation, we aim to highlight specific genetic and molecular changes that occur in response to one’s diet, notably in those suffering from obesity and diabetes. Furthermore, this review intends to connect these findings to the potential prospect of personalized therapies using (epi)genomic technology. By exploring the various genetic processes used today in clinical settings, we hope to provide an exciting opportunity into applied targeted interventions to improve patient outcomes.

## 2. Socioeconomics and Health

Socioeconomics is defined by a multitude of social and economic factors that govern a person’s everyday life. From education level, income, occupation, and diet, all these elements have direct and indirect impacts on an individual’s lifestyle. To better classify these factors, one can refer to this grouping as socioeconomic status (SES) [11]. For example, an individual with high SES is more likely to have a higher income and educational standing than their counterparts. Many studies have highlighted the importance of SES and its relationship to health and how it can be utilized as an additive tool for medical diagnosis and treatment. Compared to those with high SES, it was seen that low SES individuals suffer from poorer health outcomes and earlier mortality rates [12,13,14].

Low SES can predispose an individual to a wide range of negative health outcomes. The longitudinal study done by Steptoe et al. found that low SES accelerated aging and increased susceptibility to diabetes, coronary heart disease, and mental illnesses like depression and anxiety [15]. One proposed mechanism behind this phenomenon is that SES has been implicated in dysregulated stress hormone levels. Cohen et al. assessed 193 subjects with differing SES and found that those with lower SES had significantly higher amounts of cortisol and epinephrine and slightly higher norepinephrine [16]. Increased levels of cortisol and epinephrine, both known stress markers, can further exacerbate biological aging and the development of noncommunicable diseases like obesity and hypertension [17].

Low SES can also have profound effects on chronic wound development and healing [18]. In addition to increased susceptibility to metabolic diseases, specific factors like diet and lifestyle can also deter the wound-healing process. Food purchasing data from urban Chicago showed that individuals with low SES were more likely to purchase less healthy foods than those with high SES [19]. The proposed effects of nutrition on inflammation and wound healing are well studied and will be expanded upon later. Regarding lifestyle choices, low SES has been associated with higher rates of cigarette smoking and other risk-taking behaviors, with low educational attainment being a primary contributing factor [20,21]. Cigarette smoking has long been established as a serious health detriment. In the body, smoking tends to damage blood vessels and disrupt the capillary flow, prompting ischemia and reducing oxygen delivery, which can severely impede the healing process [22].

**Figure 1:**
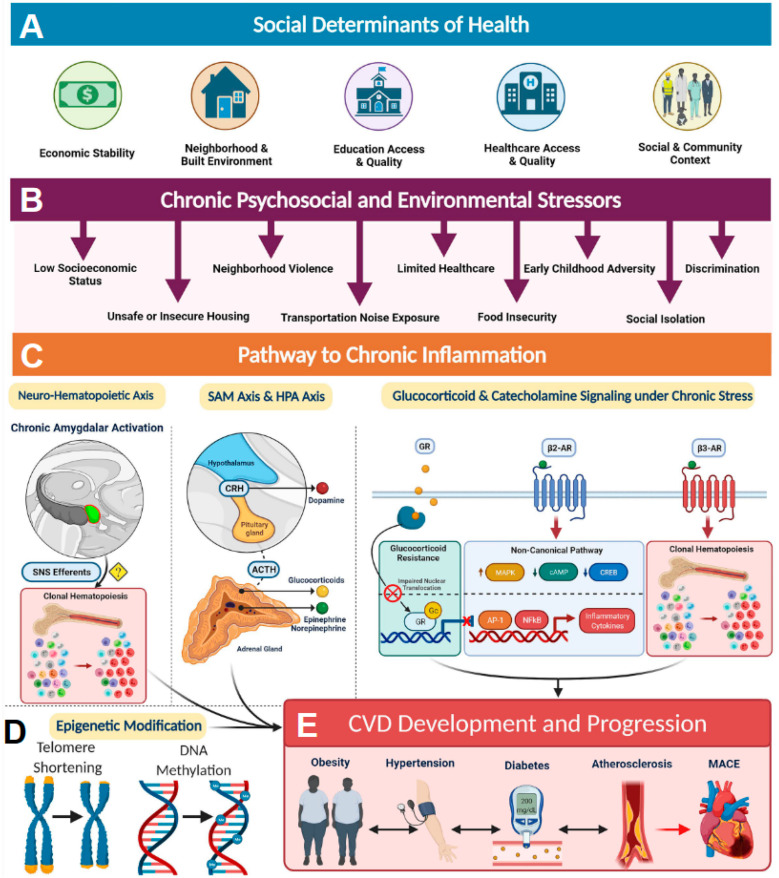
The social determinants of health and the biology of adversity. (**A**) Social determinants of health encompass an individual’s economic stability, neighborhood and built environment, education access, healthcare access, and social and community relationships. (**B**) These areas can be sources of chronic psychosocial stressors to individuals who suffer from low socioeconomic status, unsafe housing, neighborhood violence, limited access to healthcare, early childhood adversity, discrimination, increased noised exposure, food insecurity, and decreased sleep quality, among others. (**C**) Pathway to chronic inflammation: The biologic consequence of adversity promotes pathways to chronic inflammation. Sympatho-adrenomedullary (SAM) axis and hypothalamic-pituitary-adrenal (HPA) axis: the SAM axis and the HPA axis are activated by psychosocial stress and regulate the production of catecholamines (dopamine, norepinephrine, and epinephrine) and glucocorticoids, respectively. Glucocorticoid and catecholamine signaling under chronic stress: (1) Glucocorticoid receptor (GR) shows impaired nuclear translocation and decreased anti-inflammatory gene transcription in chronic stress, and (2) β-Adrenergic receptors (ARs) have been found to alter their gene signaling to a noncanonical pathway (via β-arrestin 2 scaffolding) that increases production of inflammatory cytokines, which also upregulate NLRP3 (NLR family pyrin domain-containing 3) inflammasome activity. β3 Receptors have also been found to play a role in clonal hematopoiesis, which may contribute to atherosclerotic plaque formation. Neurohematopoietic axis: Chronic amygdalar activation has been linked to clonal hematopoiesis, possibly by direct sympathetic nervous system (SNS) innervation of the bone marrow; stress-induced leukopoiesis has been directly linked to atherosclerotic plaques. (**D**) SDoH-influenced chronic inflammation often results in the accumulation of pathological epigenetic modifications and shorting of telomere length. (**E**) All of these inflammatory processes lead to increased cardiovascular disease (CVD) risk factors, such as obesity, hypertension, diabetes, and atherosclerosis, ultimately contributing to major adverse cardiac events (MACE) and CVD mortality. ACTH indicates adrenocorticotropic hormone; AP-1, activating protein-1; CREB, cAMP response element-binding protein; CRH, corticotropin-releasing hormone; MAPK, mitogen-activated protein kinases; NF-κB, nuclear factor κ-light-chain-enhancer of activated B cells; and SNS, sympathetic nervous system. Reproduced under the terms of the Creative Commons CC BY license published by Wolters Kluwer. The following original report is credited: Tiffany M Powell-Wiley et al. [14].

Social determinants of health (SDoH) account for SES in combination with other environmental and psychosocial factors that influence health and can play significant roles in disease development and progression [14]. SDoH factors are influenced by structural determinants of health or policies and systems put in place that dictate access to these resources (Figure 1A). For example, access to quality education and quality healthcare are structural determinants of health equity that are greatly influenced by SES (Figure 1A). Low SES, in combination with other SDoH factors, such as food insecurity, unsafe or insecure housing, neighborhood violence, limited medical accessibility, early childhood adversity, social isolation, and discrimination, can serve as a source of chronic stress that promotes a proinflammatory state resulting in health disparities (Figure 1B). For example, those with low SES are more likely to have lower incomes and live in disadvantaged communities, and their access to healthcare resources is limited, leading to diminished overall health [13,14] (Figure 1A,B). Elevated markers of inflammation such as CRP (C-reactive protein), sICAM-1 (soluble intercellular adhesion molecule-1), IL-6 (interleukin 6), and MCP-1 (monocyte chemoattractant protein 1) are often associated with health disparities observed in individuals exposed to chronic psychosocial stress and environmental stressors [14]. It has been established that these stressors activate the sympathetic nervous system (SNS) (Figure 1C). Activation of SNS axes such as sympatho-adrenomedullary (SAM) and the hypothalamic-pituitary-adrenal (HPA) then increases the levels of stress-related hormones such as cortisol and catecholamines among others, resulting in chronic inflammation [23]. In addition, chronic activation of SNS and HPA axes also results in glucocorticoid receptor resistance, thereby blunting anti-inflammatory response [24] (Figure 1C). Such SDoH-influenced chronic inflammation often results in the accumulation of pathological epigenetic modifications and shorting of telomere length in individuals living in disadvantaged neighborhoods and has shown alteration of gene expression, particularly among genes involved in inflammatory pathways (Figure 1D). All these inflammatory processes lead to an increased risk of developing obesity, hypertension, diabetes, and atherosclerosis, ultimately contributing to major adverse health outcomes (Figure 1E). Hence, it is important to recognize that SDoH constructs contribute to biologic adversity experienced by an individual. Incorporation of SDoH-informed screening tools into clinical settings has the potential to significantly improve patient outcomes.

## 3. Genomics and Health

Genomics is the interdisciplinary study of the structure, function, and evolution of the human genome. Understanding and applying the field of genomics is essential in scientific research. By incorporating genomics, scientists can pinpoint on a molecular level how the human body interacts with the environment [25]. Genomics can provide valuable insight into various aspects of human health, including disease susceptibility and anticipated reactions to medicine. In obesity, for example, genomic studies have linked more than 300 single-nucleotide polymorphisms (SNPs) to phenotypes like BMI and adipocyte distribution [26]. Cancer diagnosis and therapy have also been influenced by genomics. The utilization of gene sequencing has allowed clinicians to better study the development and prognosis of tumors in cancer patients, providing for improved care and health outcomes [27]. The emergence of genomics has revolutionized the healthcare field. With precise access to a patient’s biological makeup, the feat of personalized medicine has become even more attainable.

### 3.1. Sociogenomics

Sociogenomics is the study of the social and environmental processes that influence the genome [28]. With the help of recent advances in modern molecular techniques, socioeconomic factors have been documented to play significant roles in the regulation of the genome and the modification of the epigenome [18,29,30]. A study by Idaghdour et al. found that socioeconomic differences between urban and rural environments accounted for 10 times more variation in gene expression than did differences due to familial lineage and gender [31]. While not explicitly identifying the genomic deviances, this study is significant as it highlights the measurable impact one’s environment has on one’s genetic framework.

Lifestyle choices, nutrition, and social interactions are just some of the environmental factors that have been studied to alter gene expression. Archie et al. documented that the social status of wild baboons can predict outcomes of the wound healing process, with higher-ranking males healing significantly faster than lower-ranking males; proposed mechanisms involved varying effects of glucocorticoids depending on the individuals’ social status, suggesting that energetic stress of alpha males promotes wound healing whereas social stress of lower males might cause negative effects of stress to occur [32]. In humans, traumatic events have been shown to fundamentally affect the expression of genes and alter downstream biological functions through epigenetic modifications of DNA, causing long-term impacts on the neurological, endocrine, and immune systems of the affected individual [33]. In the endocrine system, it has been shown that early exposure to trauma and high-stress environments has marked effects on the regulation of the hypothalamus and pituitary gland axis [34], increasing susceptibility to metabolic stress and noncommunicable diseases [35]. Nowacka-Chmielewska et al. demonstrated that a combination of a Western diet and chronic stress environment impacted the rat brain proteome by down-regulating proteins involved in neurotransmitter secretion and learning/memory while up-regulating a protein involved in insulin-secretion in the brain, all of which may functionally impair important neuronal processes and signaling [36].

### 3.2. Genomic Wide Association Studies (GWAS) in Diabetes and Wound Healing

GWAS are genetic studies that analyze and compare individuals against a genomic database associated with a range of traits, conditions, and diseases. GWAS uses genetic markers like SNPs to identify DNA sequences that can contribute to an individual’s heightened disease risk [37]. A typical published pipeline for designing a GWAS study starts from the identification of a disease cohort and an appropriate control cohort (in this review, we take nonhealing wounds as an example; Figure 2①). The next step is to generate robust statistically tested genotype data to investigate whether an allele is associated with a disease phenotype with a usually high P-value threshold (less than ~5 × 10^−8^–5 × 10^−10^; Figure 2②). GWAS analysis next relies on the calculation of linkage disequilibrium (LD) analysis to infer the association between DNA variants and the trait to identify causal and proxy genetic variants (Figure 2③). Proxy variant is the marker that is associated with a disease phenotype but may not be the actual causal variants. Next, genome-wide Manhattan plots are generated that display GWAS findings with respect to their genomic positions, highlighting signals of known or novel association to a particular disease type (Figure 2④). Finally, additional strategies such as fine-mapping, functional confirmation, and meta-analysis are performed for bias-free identification of causal variants and to search for additional susceptibility variants (Figure 2⑤). Scientific researchers have utilized GWAS in large population cohort studies to prove the genetic significance of disease risk between a population of interest and healthy controls. For example, GWAS databases like Axiom Precision Medicine Diversity Research Array (PMDA) use familial and environmentally based SNP markers to help provide insight into a multitude of disease susceptibilities as well as alterations in drug metabolism genes [38]. However, it is important to note that GWAS is only used to establish disease association and not complete causation.

In diabetes, GWAS has been used to investigate patient susceptibility and disease severity. A study done by DeForest et al. identified 700 gene loci associated with increased risk of type II diabetes mellitus (T2DM) development [39]. Many of these loci are ancestrally based, i.e., the loci have been preliminarily scanned through a genetic database consisting of diverse populations from areas like Europe, Africa, and Asia. This adds to GWAS validity, as it incorporates genetic variations across groups of people with diverse historical backgrounds [39].

**Figure 2:**
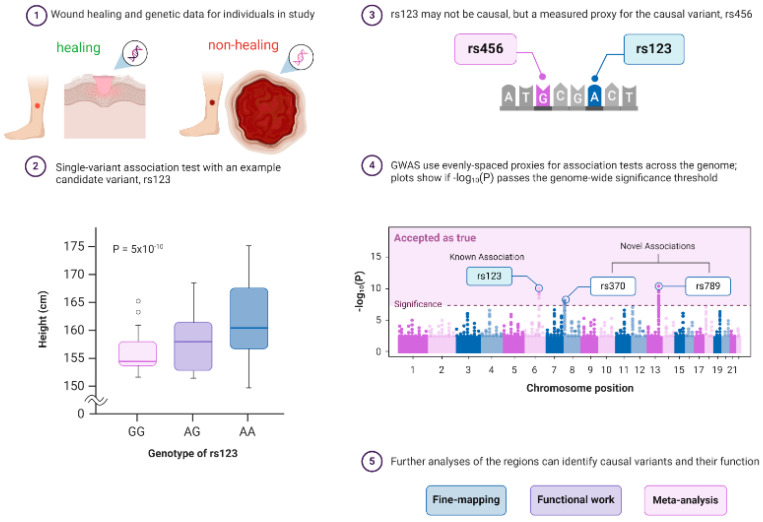
Principle of a genome-wide association study (GWAS) in wound healing research. GWAS is a hypothesis-free way of identifying genome regions associated with a trait and requires data on genetics and a trait of interest for individuals with healing wounds vs. nonhealing chronic ulcers from a population. GWASs consist of single-variant association tests for variants across the genome, but a significant association only means that a proxy for a causal variant was found. ① GWAS can confirm prior and identify novel associations, and post-GWAS analyses can determine how the regions affect the rate of healing of wounds. The typical pipeline of GWAS consists of ② the identification of a statistically significant variant, ③ testing if the associated variant is causal or just a proxy, and ④ displaying the GWAS findings with respect to their genomic positions, highlighting signals of known or novel association to a particular disease type using Manhattan plots. ⑤ Finally, additional strategies such as fine-mapping, functional confirmation, and meta-analysis are performed for bias-free identification of causal variants and to search for additional susceptibility variants. The following sources are acknowledged: Bailey Harrington (Creator) Nima Vaezzadeh, Visscher, et al. [42], and McCarthy et al. [43]. Created with BioRender.com.

Regarding wound healing, GWAS can be used to identify alterations in genes that are involved in inflammatory and healing stages (Figure 2). One valuable association SNP marker is fibrosing disorders. In fibrosis, the inflammatory process tends to be prolonged, leading to the potential formation of chronic wounds [40]. GWAS has been used to detect patients who are susceptible to fibrotic diseases, with specific loci conferring an increased risk in phenotype presentation [41]. With the help of GWAS, patients who are at risk of mutations in the healing cascade can be identified and appropriately managed.

### 3.3. Epigenetics

Recent advancements in epigenetic research have further shed light on the importance of socioeconomic factors on health. From financial status to everyday lifestyle choices, these elements have been seen to influence heritable changes in gene expression without changing the DNA sequence itself [44,45,46]. These genetic alterations can have marked impacts on health outcomes, including elevated sensitivity to obesity, sepsis, and diabetes [47,48,49,50]. A review summarized by Ling et al. found that in patients with T2DM, elevated levels of DNA methylation were seen in adipocyte-associated genes that may have relevance to the development and prognosis of diabetes [51]. Epigenetic research has the potential to innovate the healthcare system. With targeted epigenetic analysis and therapies such as gene-specific DNA demethylation [29], scientists and clinicians can approach disease processes differently and provide personalized interventions to enhance patient care [52].

## 4. The Process of Physiological Wound Healing

The four fundamental phases of the wound-healing process are hemostasis, inflammation, proliferation, and remodeling [53]. Shortly after injury to the skin tissue, platelet aggregation occurs, and the coagulation cascade forms a blood clot through the release of chemokines and growth factors to achieve hemostasis. Neutrophils and macrophages respond to inflammatory cytokines and infiltrate the injured site, cleansing the area of debris, bacteria, and damaged tissue via phagocytosis, thereby causing inflammation [54]. Fibroblasts, keratinocytes, and endothelial cells are among those cells that begin to accumulate and interact with each other during the proliferative phase of wound healing [55,56]; these cells produce the extracellular matrix, proteoglycans, collagen, elastin, and other connective tissue components that form the granulation tissue of the wound. In addition, various cytokines and growth factors are released during this proliferative phase to regulate wound healing. Finally, during the remodeling phase, some existing cells undergo apoptosis, new cells are produced, type III collagen is replaced by type I collagen, and the profuse extracellular matrix is gradually degraded. The remodeling phase is especially important in the pathogenesis of wound healing, as any errors may result in excessive inflammation and chronic wound formation [57].

It is important to differentiate between the presence of an acute or chronic wound, as each carries its respective assessment and management processes. In general, chronic wounds are long-lasting injuries that fail to fully progress through the healing process and/or are arrested in a pro-inflammatory stage. For a wound to be classified as chronic, certain guidelines have been published: (i) healing time lasting greater than 6 weeks, (ii) dysregulated inflammation or cell migratory stages, and (iii) disruption of blood flow to the wound site [58]. Chronic wounds fall under three major categories: leg ulcers, diabetic ulcers, and pressure ulcers [22]. Once a chronic wound has been identified, appropriate care must be implemented to achieve maximal healing to avoid amputation or other severe outcomes.

### 4.1. Genetics and Molecular Mechanisms of the Wound Healing Process

There are many genes and protein products involved in the wound healing process, with many playing various roles throughout the phases of healing. While this is far from an exhaustive list of every gene with a function related to wound healing, the following descriptions of the wound healing process include several genes and protein products that play significant roles in wound repair.

#### 4.1.1. Genetics and Molecular Mechanisms in the Hemostasis Phase of Wound Repair

Following the injury to the vascular endothelium, the affected blood vessel constricts, and hemostasis occurs to close the endothelium with a clot. This process of hemostasis can be divided into two types: primary and secondary. During primary hemostasis, von Willebrand Factor (*vWF*) binds to the exposed endothelium; platelets then adhere to the vascular wall via binding *vWF* with glycoprotein (*GP1b*) receptors on the platelet surface. Subsequently, *GPIIbIIIa* receptors on the platelet surface bind circulating fibrinogen; platelets secrete dense granule contents that bind purinoceptors *P2Y_1_* and *P2Y_12_* to further activate and attract other platelets, resulting in a positive feedback loop to form the beginning of a platelet plug. Additional prostaglandins from platelets, such as Prostaglandin E2 (*PGE_2_*) and Thromboxane (*TxA_2_*), are produced following Cyclooxygenase 1 (*COX-1)*-mediated oxidation of arachidonic acid to further activate platelets [59]. Secondary hemostasis consists of the coagulation cascade forming a fibrin clot via activation of circulating coagulation factors (proteins primarily produced by the liver). The intrinsic pathway of the coagulation cascade begins with the activation of factor XII and ultimately results in thrombin-mediated activation of fibrin. The extrinsic pathway of the coagulation cascade begins with tissue factor-activated factor VII complex activation of factor X, with subsequent reactions resulting in thrombin-mediated activation of fibrin to form the fibrin clot [59,60].

#### 4.1.2. Genetics and Molecular Mechanisms in the Inflammation Phase of Wound Repair

Once circulating leukocytes infiltrate damaged tissue, they begin releasing pro-inflammatory cytokines; among these, interleukin-1 (*IL-1*), interleukin-6 (*IL-6*), and tissue necrosis factor (*TNF*) play key roles in the inflammatory process. *IL-1* acts as a pyrogen for leukocytes, induces fever, and plays a role in the activation of lymphocytes [61]. *IL-6* is involved in the production of acute phase reactants by the liver, regulation of circulating iron and zinc, promotion of megakaryocyte maturation, and differentiation of naïve T and B cells [62]. *TNF* is primarily produced by macrophages and T cells; its functions are to stimulate the production of nitric oxide (NO) and lead to vasodilation, to increase vascular permeability and cause edema, to stimulate the secretion of selectins that leukocytes bind, to aid in the production of reactive oxygen species (ROS) by neutrophils during the respiratory burst, and to activate the thalamus to induce fever [63].

The regulation of inflammation is a necessary response, as prolonged inflammation can disturb the normal healing process and lead to the development of chronic wounds [64]. As the immune system recognizes the completion of the inflammatory cascade, macrophages and immune cells are induced to release anti-inflammatory cytokines such as interleukin-10 (*IL-10*), interleukin-4 (*IL-4*), and transforming growth factor beta (*TGF-β*) to counter the effects of pro-inflammatory molecules [65]. Of all anti-inflammatory molecules, *IL-10* has been identified as a powerful regulator of inflammation, embodying various roles in pain response and wound healing. A research study conducted by King et al. showed that in *IL-10* transgenic knock-out mice, sustained inflammatory response hindered the regeneration of inflicted wounds and resulted in chronicity [66].

#### 4.1.3. Genetics and Molecular Mechanisms in the Proliferation Phase of Wound Repair

During the proliferation phase of wound healing, fibroblasts and myofibroblasts produce collagen III to form granulation tissue, induced by transforming growth factor-β (*TGF-β*) and inhibited by interferon-γ (*IFN-γ*). *IFN-γ* is secreted by lymphocytes (primarily helper T cells and natural killer cells) and plays a role in the activation of immune cells, the recruitment of neutrophils, the apoptosis of damaged cells, and the enhancement of matrix metalloproteinase-9 (*MMP-9*); defects in *IFN-γ* have been shown to result in a prolonged neutrophil response and enhanced *MMP-2* activity [67]. Angiogenesis occurs due to the secretion of various factors and their receptors by macrophages and fibroblasts within the wound tissue. Such angiogenesis-promoting factors include *TGF-β*, vascular endothelial growth factor (*VEGF*), platelet-derived growth factor (*PDGF*), endothelial growth factor (*EGF*), fibroblast growth factor (*FGF*), angiogenin, angiopoietin, and human mast cell tryptase [68].

#### 4.1.4. Genetics and Molecular Mechanisms in the Remodeling Phase of Wound Repair

The remodeling phase of wound healing can last up to years. A critical component of this period is the regression of newly formed blood vessels to create a more organized, typical underlying vascular system via decreased release of pro-angiogenic factors (*VEGF* and *FGF*) and increased secretion of anti-angiogenic factors (*PEDF* and *SPRY2*) and maturation factors (*ANG1* and *PDGF*) [69]. In addition, collagen remodeling occurs as *MMP-2* and other collagenases are upregulated to degrade the type III collagen produced during the proliferation phase, which is later replaced with a more mature type I collagen [70,71]. Disruptions to any of these processes can lead to scar formation and an increased likelihood of developing a chronic wound.

## 5. Impact of Socioeconomic Status on the Healing Process

Recent studies have suggested that socioeconomic factors can influence how the body responds to inflammatory stimuli. From cytokine release to leukocyte migration, altered expression of these responses can have dramatic effects on wound healing (Table 1). In a genomic study of individuals experiencing chronic social isolation compared to those who are socially connected, Cole et al. found significant results showing the upregulation of pro-inflammatory mechanisms in socially isolated subjects [72]. Transcription factors *NF-κB* and *AP-1*, which produce pro-inflammatory cytokines like *IL-6* and *TNF*, were found to have increased activity, while glucocorticoid receptors, which are known to have anti-inflammatory properties, had decreased activity. A related study looking at mammalian childhood adversity and stressful upbringing likewise saw associated impacts on pro-inflammatory processes, as well as altered leukocyte gene expression [73].

### 5.1. Pathophysiological Mechanisms of Altered Gene Expression in Chronic Wounds

#### 5.1.1. The Conserved Transcriptional Response to Adversity (CRTA)

One theory explaining the mechanism by which social and environmental factors alter gene transcription levels is known as the CRTA. The CRTA states that in times of chronic environmental stress, the central nervous system’s perception of perceived adversity can stimulate the constant release of norepinephrine that modifies the sympathetic nervous system; this may alter levels of secondary messenger systems, raise the activity of pro-inflammatory pathways via amplified inflammatory leukocyte production and cytokine gene transcription (*NF-κB*, *IL-1*, *IL-6*, *IL-8*, *COX-2*, *TNF*), increase risk of infection via decreased lymphocyte production, and ultimately, predispose one to a variety of cancers and chronic disease [80]. The chronic inflammatory state seen in such circumstances can have a profound impact on an individual’s health and risks of disease states; among these is the risk of developing a chronic wound.

#### 5.1.2. Diabetes and Impairment of Wound Healing

In 2018, the CDC estimated that over 34 million people were suffering from diabetes in the US, with the cost of care accounting for almost 1 in 4 total US healthcare dollars; trends in medicine have suggested that cost and incidence rates are only looking to increase [81]. Diabetes poses numerous health dangers, including patients having an increased risk of chronic wound development. Sustained hyperglycemia associated with diabetes poses the most significant pathophysiological mechanism behind altered wound healing in diabetic patients [30,82,83]. As patients have increased serum glucose, blood vessels supplying tissues become stiffer and necrotic, leading to hypoxia, disrupted angiogenesis, and impaired immune cell migration [84]. Complications of vascularity can predispose diabetic patients to poor wound healing and lead to the development of chronic wounds, specifically diabetic foot ulcers, that can have significant impacts on patient livelihood. It is estimated that 15% of people with diabetes develop diabetic foot ulcers, and 5–8% of patients with a diabetic foot ulcer will require amputation [85].

Diabetic wounds exhibit cellular dysfunction in all steps of the wound-healing process. Endothelial/epithelial cells have decreased *PDGF* receptor expression, causing a prolonged transition period between hemostasis and inflammation; the inflammatory phase is lengthened due to an increase in the number of macrophages present in wounds, with a secondary increase in expression of numerous inflammatory cytokines [86,87,88]; and the proliferation and remodeling phases are impaired via inadequate fibroblast signaling [89,90], delayed keratinocyte migration and re-epithelization, increased expression of MMPs and reactive oxygen species, and dysfunctional *VEGF* signaling blunting angiogenesis [91,92,93]. Mechanisms behind transcriptional, proteomic, and cellular changes in diabetic tissue vary. For example, Wang et al. demonstrated that two miRNAs that target and degrade specificity protein-1, which binds the *MMP-9* promoter and enhances transcription—are significantly downregulated in diabetic tissue, thereby leading to an overexpression of *MMP-9* [94]. Oxidative stress in vascular endothelial cells due to hyperglycemia has been noted to induce the transcription of *MMP-9*, which can generate damage to healing tissue and lead to poor wound healing [95]. Diabetes fundamentally alters the tissue environment, thereby modifying many important molecular pathways involved in proper wound repair.

## 6. Impact of Nutrition on the Healing Process

In addition to disrupted social environments, the effects of poor diet have also been well documented in the inflammatory response. With less access to proteins and micronutrients, intestinal dysbiosis can occur, leading to positive feedback of inflammatory mechanisms [96,97]. Patterson et al. found that protein-deficient mice had exaggerated systemic inflammation at baseline, as well as heightened response to inflammatory stimuli [98]. Many dietary, lifestyle, and health factors have been demonstrated to affect the wound healing process and ensure varying healing statuses in patients. From a nutritional perspective, macromolecule levels, vitamin and mineral stores, exercise, obesity, and diabetes are among the most important social factors impacting wound healing [99].

### 6.1. Specific Genes and SNPs Associated with Nutritional Pathology and Poor Wound Healing

With the advancement in the fields of nutrigenetics and nutrigenomics, it is now clear that genetic variants affect the intake of available nutrients [100]. Genomic diversity in ethnic groups influences the bioavailability of nutrients and their metabolism [100]. In the future, such gene-nutrient interaction could lead to the development of personalized nutritional recommendations for patients with chronic wounds. The following section investigates the genetic changes in important genes that may lead to nutritional pathologies.

#### 6.1.1. MTHFR

The *MTHFR* gene (NCBI—Gene ID: 4524) encodes the methylenetetrahydrofolate reductase enzyme that catalyzes the conversion of 5,10-methylenetetrahydrofolate to 5-methyltetrahydrofolate, a co-substrate for homocysteine re-methylation to methionine. Polymorphisms in the *MTHFR* gene can cause hyperhomocysteinemia, which may have wide-ranging impairing effects on the wound-healing process [101]. Vasculopathies, thromboses, and vascular ulcers due to blood clots caused by high homocysteine levels in the blood may impair the coagulation system, thereby altering proper hemostatic function [102,103]. Individuals with a homozygous mutant substitution in *MTHFR* have been shown to have elevated C-reactive protein (CRP), fibrinogen, and white blood cell (WBC) count, suggestive of systemic inflammation related to hyperhomocysteinemia [104]. High plasma homocysteine levels may decrease nitric oxide (NO) activity within wounds and possibly modify granulation tissue deposition during wound matrix formation, thereby impairing proper inflammatory and remodeling phases of wound healing and leading to the development of chronic wounds [105].

Frosst et al. identified one SNP in the *MTHFR* gene, *rs1801133*, an *A222V* substitution, that was linked to having reduced enzyme activity, increased thermolability of enzyme from lymphocyte extracts, and significantly elevated homocysteine levels in plasma [106]. In some patients with chronic wounds and elevated homocysteine levels, administration of oral supplemental vitamins such as folate, pyridoxine hydrochloride, and cyanocobalamin has been shown to be beneficial and promote proper wound healing [103,105]. This raises the question of whether it is possible to bypass altered gene function due to *MTHFR* gene mutation (i.e., *rs1801133*) via dietary supplementation of vitamins involved in the same metabolic pathway.

#### 6.1.2. VDR

The *VDR* gene (NCBI—Gene ID: 7421) encodes the vitamin D3 receptor, with downstream targets involving mineral metabolism, the immune response, and cancer. This gene is vital to proper wound healing and has been described as a “master regulator of tissue damage and acute inflammation” due to the heavy involvement of the vitamin D3 signaling pathway in skin barrier function, inflammation, microbial defense, and stem cell growth [107]. Experiments in mice have revealed that deletion of *VDR* from keratinocytes in combination with a low-calcium diet significantly delays skin wound healing, likely due to the reduction of β-catenin signaling in proliferating cells at the edge of wounds that are dependent on proper *VDR* and calcium signaling [108]. *VDR* knockout or vitamin D-deficient mice have reduced corneal epithelial wound healing, regardless of supplementation with calcium, lactose, and phosphate [109]. Patients with leg ulcers are more likely to have vitamin D deficiency, with a trend toward better healing in those with vitamin D supplementation [110]. Vitamin D deficiency has been proposed to be a marker of comorbid illness for patients with pressure ulcers [111] and negatively impacts treatment outcomes post-periodontal surgery [112]. In patients with diabetes, having a vitamin D deficiency is associated with difficulty healing or having stalled/deteriorating wounds [113]. Additionally, vitamin D supplementation has been shown to have beneficial effects on glycemic control, inflammatory marker levels, and cholesterol levels in patients with diabetic foot ulcers [114].

The *rs1544410* SNP of the *VDR* gene has been associated with increased insulin secretion in women with post-gestational diabetes mellitus, suggesting a preventive role in developing post-gestational diabetes mellitus [115]. The same SNP has also been significantly linked to adiposity phenotype indicators (BMI, waist circumference, and body fat percentage) associated with obesity [116]. As previously noted, diabetes and obesity have been associated with poor wound outcomes; therefore, *rs1544410* may play both a beneficial (in the case of diabetes development) and a detrimental (in the case of obesity) role in wound outcomes based upon nutritional-pathologies.

#### 6.1.3. MMP-9

*MMP-9* gene (NCBI—Gene ID: 4318) encodes the enzyme matrix metalloproteinase 9, which is responsible for degrading type IV and V collagen fibers. During the wound healing process, *MMP-9* is expressed and secreted by granulocytes and skin fibroblasts in wounded tissue, with increased expression when exposed to *IL1-β*, *TGF-β1*, and *TNF-α* [117]. In mice models, *MMP-9* deficiency has been shown to impair wound neovascularization, increase inflammation (further exacerbated in diabetic mice), and reduce collagen deposition and peripheral blood endothelial progenitor cell counts [118]. Elevated levels of *MMP-9* have been associated with poorer wound outcomes, particularly in diabetic foot ulcers and venous ulcers [119,120,121]. *MMP-9^−/−^* null mice studies demonstrated that *MMP-9* plays a role in modulating cholesterol metabolism, suggesting that dysregulation of this system may cause metabolic disorder, atherosclerosis, and coronary heart disease [122]. Glucose intake has been shown to increase proinflammatory pathways, thus increasing the expression of plasma *MMP-9* [123]. These studies indicate the mutual relationship between nutritional status, metabolite levels, and *MMP-9* expression, prompting the need for further research into the effects of altered *MMP-9* expression due to changes in nutrition on the wound healing process. Targeted therapy to reduce *MMP-9* levels or inhibit expression in chronic wound patients has been hypothesized to promote a healing status [121]; the possibility that nutritional changes may impact *MMP-9* levels and promote healing in chronic wound patients warrants further investigation via randomized control trial.

The *rs3918242* SNP affects the promoter region of the *MMP-9* and has been implicated in increasing risk for myocardial infarction, lower ejection fraction, progression to left ventricular systolic abnormalities [124], multiple gingival recessions [125], ischemic stroke [126], T2DM, and diabetic foot ulcers [127]. In newborns, a decrease in birth weight was linked to the *rs17577* SNP in mothers and *rs17576* in newborns, while an increase in birth weight was linked to *rs17577* in newborns [128]. The presence of specific SNPs in the MMP-9 gene may alter the gene function significantly enough to induce metabolic changes that could impact the development or prognosis of chronic wounds.

## 7. Macronutrients and Micronutrients in Wound Healing

**Figure 3:**
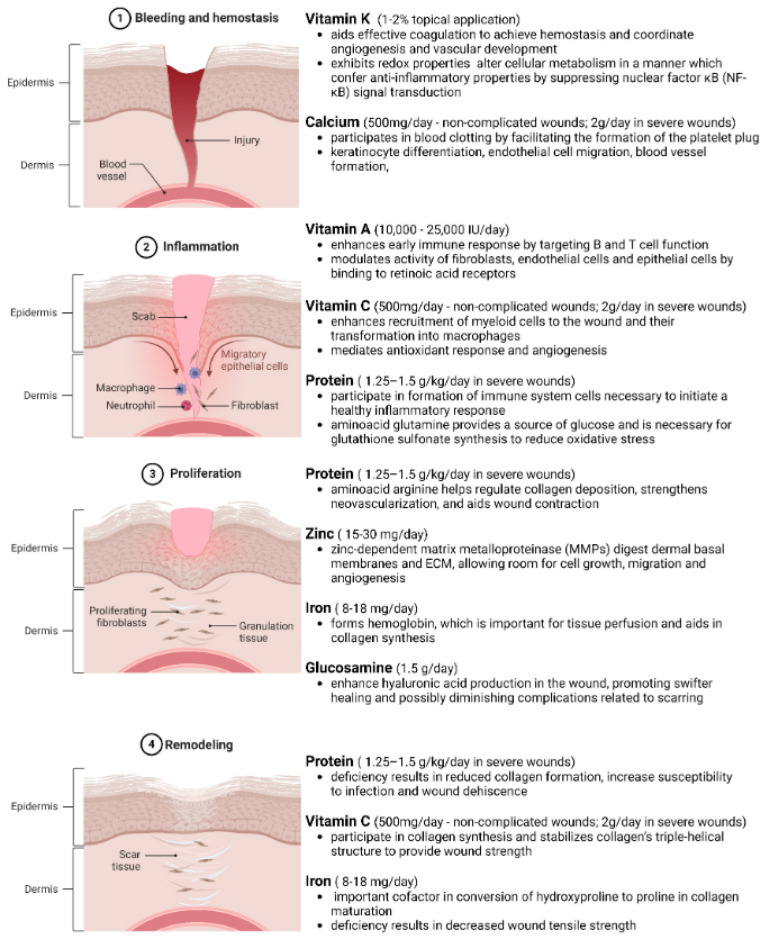
Phases of Wound Healing and Nutrient Requirements in Different Phases. Wound healing is a complex, well-orchestrated process occurring in four distinct yet overlapping phases. The process commences with ① bleeding and hemostasis, where the injury occurs, clot formation and platelet activation halt blood loss. Following this, ② inflammation sets in, combating infections and clearing debris. ③ The proliferation phase ensues, involving fibroblast activation, angiogenesis, and granulation tissue formation. ④ Lastly, remodeling occurs, where the extracellular matrix is restructured, and the tissue regains its tensile strength with the scar tissue formation. Understanding the nutritional requirements of these biological events is pivotal for developing effective wound management strategies. Adequate nutrition via sufficient macromolecule intake is necessary to meet the metabolic requirements of different phases of the healing tissue. In obese and diabetic individuals, general nutritional deficiencies have been seen due to poor-quality diets, higher intake needs, and altered cell-signaling and metabolic pathways. Created with BioRender.com.

Adequate nutrition via sufficient macro- and micronutrient intake is necessary to meet the metabolic requirements of different phases of the healing tissue (Figure 3) [129,130,131,132,133,134,135,136]. The wound healing process is divided into four integrated phases—namely, hemostasis, inflammation, proliferation, and remodeling (Figure 3). During this process, the overall metabolism increases, resulting in increased demand for nutrients and calories [137]. In chronic wounds, healing is delayed due to significant alterations in nutrient availability and metabolism. Hence, a proper nutritional supplementation guideline is necessary to address the malnutrition associated with chronic wounds. In obese and diabetic individuals, general nutritional deficiencies have been seen due to poor-quality diets, higher intake needs, and altered cell-signaling and metabolic pathways [138]. These effects can have profound limitations on the wound-healing process. After just 4 weeks of general malnutrition, immune cell functioning decreases significantly, resulting in potential infection and delayed healing response [79]. Below are examples of macronutrients and micronutrients and their effects on wound healing phenotype. The dietary factors discussed below and in Table 2 are based on the extensive review articles published on the nutritional support required for chronic wound management by MacKay et al. [139], Molnar et al. [140], Munoz et al. [129], and Barchitta et al. [133].

### 7.1. Protein

Protein, a macronutrient found readily in meat and poultry, comprises a combination of 20 amino acid building blocks. Each amino acid contributes to the protein’s unique polymer structure and function [176]. Every protein plays a distinct role in the body, with ranging effects on cellular signaling and migration to inflammatory healing [133]. Protein intake is crucial for the proper healing of wounds. Breslow et al. found that malnourished patients with pressure ulcers who are prescribed high-protein diets may have better healing than those on lower-protein diets [177]. Various amino acids have studied roles in the inflammatory and wound healing process. Proline and glycine are the building blocks of collagen. A deficiency in collagen formation can lead to wound dehiscence and impaired regeneration, as collagen is vital in the proliferation and remodeling phases of wound healing [141]. Other amino acids, like arginine and glutamine, are involved in various stages of the inflammatory cascade, including nitric oxide production, collagen synthesis, and the modulation of inflammation [143]. In times of metabolic stress, Arribas-López et al. found that arginine and glutamine supplementation at the appropriate dosages can improve wound healing at one or more stages [178]. These four amino acids—proline, glycine, arginine, and glutamine—are closely associated with critical processes in wound healing, highlighted by the aforementioned effects in tissue repair and regeneration [178,179].

### 7.2. Fatty Acids

Like proteins, fatty acids make up important cellular structures and provide necessary functions for proper wound healing [180]. There are two main categories of fatty acids present in everyday diets: saturated and unsaturated fats. Saturated fats are typically solid at room temperature and are found in foods like red meat and dairy [181]. Unsaturated fats are liquid at room temperature and are found in plant-derived foods like nuts and olive oil [182]. There is an abundance of literature on both types of fats, as they each contribute to many biochemical mechanisms in the human body, including the inflammatory process. Unlike unsaturated fats, saturated fats have been linked to impaired wound healing [183]. A diet high in saturated fats has been shown to induce a state of chronic inflammation through the release of proinflammatory cytokines, disrupting the natural progression of the inflammatory cascade [184]. In contrast, unsaturated fats, such as omega-3 and omega-6 fatty acids, have been associated with anti-inflammatory effects and enhanced wound repair. Omega-3 fatty acids, more commonly recognized as fish oil, are widely consumed, deeply researched, and proven to improve wound epithelization and reduce collagen deposition during scar formation [185]. Linoleic acid and arachidonic acid—both omega-6 fatty acid derivatives—also promote wound healing through their antioxidant effects and roles in the later stages of the inflammatory cascade by modulating and controlling excessive proinflammatory processes [167]. Appropriate and balanced intake of saturated and unsaturated fats is necessary to ensure increased energy needs from wound repair processes are met, along with the anti-inflammatory benefits they provide.

### 7.3. Carbohydrates

Adequate consumption of carbohydrates and the resulting stimulation of insulin release has been shown to be required for the proliferation and migration of fibroblasts for functioning leukocyte activity and for the release of growth factors and other hormones involved in healing tissue [133]. Like fatty acids, carbohydrates can also be separated into two subgroups based on their structural complexity: simple carbohydrates (monosaccharides and disaccharides) and complex carbohydrates (polysaccharides). Due to their elongated and bulkier structure, complex carbohydrates require longer digestive and absorptive times in the body compared to simple carbohydrates [186]. Fiber, a known complex carbohydrate found largely in fruits and vegetables, has recently been researched in wound healing [187]. In a mice study done by Canesso et al., the authors compared superficially wounded mice receiving enriched fibers like guar gum against those receiving a low-fiber diet [188]. The authors found that the mice receiving a high-fiber diet had significantly accelerated wound closure times. It was also observed that mice in the high-fiber cohort had produced greater amounts of cytokines associated with angiogenesis and collagen deposition [188]. Apart from baseline inflammatory roles, other types of complex carbohydrates, like starch and cellulose, lack research on specific wound healing roles. Interestingly, starch has been recently identified for use in nanofibrous scaffolds for wound healing due to its proposed cellular proliferation properties in tissue engineering [189]. In conclusion, as proteins, fatty acids, and carbohydrates make up a large portion of one’s diet, it is important to be cognizant of their balance and composition, particularly in the setting of wound healing. As stated earlier, excessive consumption of saturated fats has been linked to chronic inflammatory states, which can prolong and impair wound healing. A well-balanced diet is necessary to optimize wound healing and overall health [190].

### 7.4. Vitamins

All vitamins essential to the human diet have a function in wound healing [133]. As most vitamins are not synthesized naturally in the body, it is important in times of heightened metabolic stress to be cognizant of one’s micronutrient intake. Below is a description of all major vitamins and their roles in wound healing.

#### 7.4.1. Vitamin A

Vitamin A is necessary for proper lymphocyte functioning, epithelialization, collagen synthesis, and deposition of granulation tissue; deficiencies of vitamin A are known to inhibit wound healing, regardless of steroid treatment [133,191]. In instances of prolonged infection, vitamin A supplementation was proven to reduce morbidity and mortality and increase wound healing activity via the immune response [147,192].

#### 7.4.2. Vitamin B

The B vitamins are cofactors for various biochemical reactions involved in leukocyte formation, immune response, and various other processes that occur during wound healing. Mochizuki et al. found that in diabetic mice, vitamin B supplementation resulted in accelerated wound closure and improved healing outcomes [151]. In total, eight B vitamin subtypes have been identified in the human body, and many are regular participants in wound healing. Vitamin B2 (riboflavin), vitamin B6 (pyridoxine), and vitamin B7 (biotin), for example, have been shown to play various roles in energy production, cellular migration and differentiation, and collagen synthesis [193]. In isolated riboflavin, pyridoxine, and biotin-deficient rats, it was seen that excision and incision wounds contracted at a much slower rate, held significantly less collagen content, and overall had impaired wound healing phases [152].

#### 7.4.3. Vitamin C and K

The role of vitamin C has been implicated in all stages of the healing processes, having significant roles in the promotion of collagen synthesis, migration of fibroblasts, and reduced oxidative damage to epithelial tissue; deficiency of vitamin C is well-documented to be associated with poor wound healing due to inadequate collagen formation [154,194]. Vitamin K is a cofactor for γ-carboxylation reactions to synthesize several factors involved in the coagulation cascade, with evidence suggesting that topical application can promote increased wound closure [132].

#### 7.4.4. Vitamin D

As previously described, vitamin D has long been implicated in wound healing. Along with its natural anti-inflammatory effects, vitamin D has been shown to promote angiogenesis and anti-infection mechanisms, both of which are crucial in wound healing [157,158]. In a double-blind, randomized placebo-control trial, Razzaghi et al. studied the effects of vitamin D supplementation on patients with diabetic foot ulcers and found significant healing results, including reductions in ulcer length, width, and depth [114].

#### 7.4.5. Vitamin E

Compared to other vitamin types, vitamin E has not been studied to significantly affect the wound healing process. Apart from its natural antioxidant capabilities and enhancement of cellular repair, vitamin E’s role in the wound healing process remains inconclusive [195]. There are various anecdotal reviews concerning the use of vitamin E oil on healed skin wounds to diminish excessive scarring; however, researchers claim that there is insufficient evidence to support this claim [196]. Thus, while vitamin E is important in maintaining overall bodily health, it is not considered a large contributor in the wound healing process.

### 7.5. Mineral Nutrients

Of the minerals in the body, iron, zinc, and calcium play particularly important roles in wound healing [130,133]. Iron is a necessary part of hemoglobin required to transport oxygen to tissues and is required for collagen metabolism; thus, iron deficiency can cause ischemia, impaired collagen production, decreased wound strength, and poor wound healing [133,169]. Calcium plays important roles in the coagulation cascade, in modulating neutrophil activity during inflammation, in promoting proper keratinocyte function to re-epithelialize wounds, in aiding fibroblasts in the contraction of wound edges to reduce wound size, and in signaling pathways during angiogenesis [130]. Finally, zinc, a crucial mineral necessary for numerous cellular processes, has been linked to poor wound healing when a deficiency occurs; in addition, topical zinc oxide has been shown to improve wound healing rate, along with some evidence suggesting zinc supplementation results in similar outcomes [197,198].

### 7.6. Clinical Evidence of Nutritional Supplementation on Wound Healing

Apart from targeted diets to correct nutritionally deficient patients, it is important to also summarize nutritional supplementation that has been proven in the literature to enhance wound healing. The following supplements are discussed based on their proven healing capabilities in pressure ulcers and leg ulcers, both common chronic wounds particularly relevant in patients with obesity and diabetes. Inadequate nutrition is the common denominator of all chronic wound patients. In the management of pressure ulcers, the European Pressure Ulcer Advisory Panel (EPUAP) recommended a minimum of 30–35 kcal/kg of body weight [140]. Protein needs are increased in patients under metabolic stress or recovering from surgical procedures, ranging from 1.0 to 2.0 g/kg of body weight, depending on the diagnosis. In addition, the Agency for Healthcare Research and Quality (AHRQ) recommends a protein intake of between 1.25 and 1.5 g/kg of body weight and fluid intake of 1 mL/(kcal·day^−1^) based on EPUAP findings [140]. Arginine, an amino acid directly involved in nitrous oxide production and cell proliferation, has become a widely used supplement in wound care [144]. Schneider et al., in a systematic review of arginine supplementation in wound healing, found several studies proving improved wound healing in patients supplementing arginine. One such study saw that patients with protein and arginine supplementation twice daily had marked improvement in ulcer healing [199].

Vitamin C, a direct co-factor in collagen synthesis and antioxidation, has been vastly researched in patients with various wound presentations [200]. In a systematic review done by Bechara et al., the authors examined two studies that supported the supplementation of vitamin C in patients with foot and pressure ulcers [200]. In one randomized control trial looking at foot ulcers, vitamin C-supplemented patients compared to placebo patients were found to have significantly improved healing and wound closure at 8 weeks after initiation of supplementation [201]. In the pressure ulcer randomized control trial, the authors found that patients supplemented with vitamin C were seen to have an 84% reduction in pressure-sore wound area compared to 42.7% in the placebo group [202]. For Vitamin C, individuals with small wounds or pressure ulcers should consider supplementation with 500 to 1000 mg of ascorbic acid daily in divided doses for optimal utilization [140]. However, in more severe wounds, e.g., burns over a large surface area, the doses can be increased up to 2 gm [140].

Along with vitamin C, zinc supplementation has also been shown to benefit wound healing in patients with pressure injuries [203]. Zinc supplementation of up to 40 mg elemental zinc/day (176 mg zinc sulfate) for up to 10 days has been recommended to enhance wound healing if a zinc deficiency is indicated in patients with pressure ulcers [140]. A systematic review conducted by Song et al. found multiple randomized controlled trials supporting the use of zinc supplementation in patients suffering from pressure ulcers [203]. One such trial, administered by Sakae et al., showed that the supplementation of zinc-L-carnosine in patients with pressure ulcers corresponded to a significant improvement rate of ulcer healing at 4 weeks [198].

Due to their individual capabilities, numerous successful randomized trials have been conducted on patients supplementing a combination of arginine, zinc, and vitamin C for healing their ulcers [204]. A large-scale study organized by Cereda et al. found that following supplementation of arginine, zinc, and vitamin C in patients with pressure ulcers, significant reductions in ulcer area (greater than 40%) were seen [205], showing the value of nutritional supplementation in wound healing. Oral administration of vitamin A to patients at 10,000–15,000 IU/day has been suggested by Stechmiller [99] to enhance wound healing in patients receiving corticosteroids. However, Molnar et al. have recommended 20,000–25,000 IU of Vitamin A treatment as a short course of 10–14 days to be safe and used with caution in liver malfunction [140]. Administration of adequate amounts of glucosamine (1.5 gm/day) by mouth during the first few days after injury can enhance hyaluronic acid production in the wound, promoting swifter healing and possibly diminishing complications related to scarring [135,206].

## 8. Obesity, Oxygen, and Hypoxia

Obesity is a chronic disease with numerous potential contributing factors, including lack of exercise, poor diet, or even genetic predisposition. Evidence suggests that patients who are obese are in a chronic state of low-grade inflammation, harboring inflamed adipose tissue like that of chronically wounded tissue, complete with immune cell infiltration and remodeling of tissue [207]. Obese individuals have impaired adhesion, migration, and proliferation of vasculogenic progenitor cells that are important for tissue repair. Murine experiments have demonstrated a significant decrease in vasculogenic progenitor cells after wounding in obese mice, with corresponding delayed vasculogenesis and wound healing as compared to non-obese mice [208]. One of the major mechanisms behind altered wound healing in obese individuals is increased body habitus-induced hypoperfusion of tissue, resulting in hypoxic- and ischemic-related molecular changes to tissue repair. While acute hypoxia in wounds promotes the initial wound-healing process, chronic hypoxia can delay wound healing via the prolonged release of cytokines and growth factors (*PDGF*, *TGF-β*, *VEGF*, *TNF-α*) from cells within the wound tissue and cause an overproduction of reactive oxygen species [162,209]. These inflammatory and proliferative factors are vital to proper wound healing, and dysfunction results in poorly healing wounds commonly associated with obese individuals. One of the primary mediators of molecular changes in response to hypoxia is hypoxia-inducible factor-1α (*HIF-1α*); normally, *HIF-1α* is upregulated in response to ischemia, with many resultant downstream effects. However, in chronic wounds, *HIF-1α* may be deficient, resulting in a failure of the wound to upregulate transcription of *VEGF* or increase the rate of anaerobic metabolism [210]. As with diabetes, obesity—often due to a multitude of environmental and social causes—alters the microenvironment of the tissue, with accompanying molecular and cellular dysfunction prime for poor wound healing. Table 2 catalogs published macro- and micronutrients involved in different phases of wound healing that are deranged during obesity.

### Exercise, Obesity, and Wound Healing

Numerous studies on exercise have highlighted its importance in various physiological functions in the body. Through its effects on blood circulation, oxygen and nutrient delivery, and the transport of immune cells, regular physical activity has been shown to act as a catalyst in the healing process, increasing rates by as much as 25% [211]. By enhancing the inflammatory response, exercise can be utilized as an avenue for patients suffering from chronic wounds. A recent study on endorphins further emphasizes the importance of exercise on wound healing. It was found that beta-endorphins released after sufficient physical activity improve keratinocyte cellular migration and wound remodeling [212,213].

Obesity and exercise play opposing roles in the prognosis of wound healing. Through numerous established studies, both clinical and experimental, Pence et al. analyzed circumstances of exercise and chronic wounds and their influence on obesity or diabetes [214]. For patients with venous leg ulcers and diabetic foot ulcers, adhering to a structured exercise training protocol when also provided usual care was shown to improve both the rate and extent of wound healing, compared to patients receiving only usual care [215]. Patients with T2DM who were assigned to and adhered to a daily foot exercise protocol were shown to have decreased diabetic foot ulcer sizes after 3 months, as compared to control [216]. The importance of staying active and engaging in the movement of wounded areas is beneficial in accelerating the rate of healing and prevention of chronic wound enhancement; when activity level is low, and body mass index (BMI) is high, patients are at an increased risk of developing chronic wounds and having delayed wound healing. Patients with a BMI greater than 40 were found to be at a significantly increased risk of developing pressure ulcers (three times greater risk) as compared to patients with a BMI less than 40 [217]. Table 2 presents the list of macromolecules involved in wound healing processes that are deranged in obesity.

## 9. Conclusions

The field of sociogenomics is an emerging discipline in molecular biology and genetics, particularly in its implementation into wound healing research. The impact of social, environmental, and nutritional factors on pathological wound development has been well described. A proper and well-balanced diet consisting of macronutrients and micronutrients is essential to proper wound healing. Through the works of numerous studies, this review has identified that the concept of both nutritional deficiency and nutritional supplementation plays crucial roles in the wound healing process and the potential for chronic wound formation. Additionally, it has been established that nutrition-related pathologies such as diabetes and obesity are known to impair tissue repair and induce a chronic state of inflammation. Nutrition and nutritional pathologies affect the individual at the molecular and biochemical level, resulting in abnormal gene expression and protein production changes, leading to an imbalance in homeostasis. Using epigenetics and the notion of personalized medicine through the human genome, exciting new research is on the horizon. As described earlier, genotypes of wound repair genes can potentially result in different wound outcomes, dependent upon various individual nutritional, social, and environmental factors. Future research is necessary to further explore the cellular and molecular etiologies by which environmental and social factors produce alterations in gene transcription and translation that can cause abnormal wound healing while also providing promising insight into targeted medicine prospects capable of reshaping the landscape of healthcare.

## Figures and Tables

**Table 1:** Summary of Socioeconomic Factors Disparities and their Impact on Wound Healing.

Socioeconomic Factor	Low SES Predictor	Impact on Chronic Wounds
Social Status	Prolonged social isolation [74]	Upregulation of pro-inflammatory mechanisms; disruption of wound healing stages [64,72]
	Childhood adversity [75]	Heightened pro-inflammatory processes, altered leukocyte gene expression; disruption of wound healing process [64,73]
Financial Status	Lower income—diminished access to healthcare [76]	Chronic wound susceptibility and aggressiveness [13,77]
Nutritional status	Poor diet and malnutrition [78]	Delayed and improper wound healing at all stages [79]
Educational Status	Lower educational attainment—increased risk-taking behaviors like cigarette smoking [21]	Bodily health detriment, ischemic consequences in wound healing [20,22]

**Table 2:** List of Derange Wound-Related Macro- and Micronutrients in Obesity.

Macronutrients/Micronutrients	Major Wound Healing Functions	Obesity; Negative Consequence
Amino Acids		
Proline and Glycine	Building blocks of collagen, wound proliferation, and remodeling stages [70,141]	Decreased serum levels; wound dehiscence and impaired regeneration [71,142]
Arginine and Glutamine	Cell proliferation, nitric oxide (NO) and collagen synthesis, angiogenesis, inflammation control [143,144]	Abnormal cellular metabolism and signaling pathways; impaired wound-breaking strength [145,146]
Vitamins		
Vitamin A	Epithelization and fibroplasia, anti-infection properties [147,148]	Impaired signaling pathways in tissues and organs; associated with increased wound infection susceptibility [149]
Vitamin B	Energy production, red blood cell synthesis, co-factor in various metabolic pathways [150]	Decreased serum levels; delayed wound contraction, remodeling, and impaired regeneration [151,152,153]
Vitamin C	Key role in collagen synthesis, maturation, and secretion, antioxidant properties [154]	Serum levels are inversely related to BMI; impaired proliferation and remodeling stages; decreased angiogenesis [155,156]
Vitamin D	Anti-infection properties, epidermal growth factors, angiogenesis [157,158]	Strongly correlated deficiency, polymorphisms in vitamin D receptor (VDR) gene; systemic inflammation and impaired healing [159,160,161]
Vitamin E	Anti-inflammatory and antioxidant properties, cellular membrane integrity [162]	Decreased serum levels; can prolong inflammatory response and lead to excessive scarring [163]
Vitamin K	Significant role in the blood coagulation cascade [164]	Decreased serum circulatory levels; reduced clotting in inflammatory response, delayed healing [165,166]
Fatty Acids		
Omega-3 and Omega-6 fatty acids (unsaturated fats)	Roles in all phases of wound healing, anti-inflammatory processes [167]	Increased levels; can induce chronic systemic inflammation [168]
Minerals		
Iron	Oxygen delivery, collagen synthesis, epithelization processes [169]	Disruption of iron homeostasis; ischemic consequences in wounds [170]
Calcium	Fibroblast and keratinocyte migration and proliferation; important coagulation factor [130]	Abnormal calcium metabolism; delayed wound healing and chronicity formation [171,172]
Zinc	Angiogenesis, tissue re-epithelization, immune and inflammation response [173]	Serum deficiency; delayed wound healing, reduced mitotic activity and scar maturation [174,175]

## Data Availability

No data were used to support this review article.
